# Non-conscious processes in changing health-related behaviour: a conceptual analysis and framework

**DOI:** 10.1080/17437199.2015.1138093

**Published:** 2016-02-16

**Authors:** Gareth J. Hollands, Theresa M. Marteau, Paul C. Fletcher

**Affiliations:** ^a^ Behaviour and Health Research Unit, University of Cambridge, Cambridge, UK; ^b^ Department of Psychiatry, University of Cambridge, Cambridge, UK

**Keywords:** Health behaviour, non-conscious, awareness, automatic, intervention, behaviour change

## Abstract

Much of the global burden of non-communicable disease is caused by unhealthy behaviours that individuals enact even when informed of their health-harming consequences. A key insight is that these behaviours are not predominantly driven by deliberative conscious decisions, but occur directly in response to environmental cues and without necessary representation of their consequences. Consequently, interventions that target non-conscious rather than conscious processes to change health behaviour may have significant potential, but this important premise remains largely untested. This is in part due to the lack of a practicable conceptual framework that can be applied to better describe and assess these interventions. We propose a framework for describing or categorising interventions to change health behaviour by the degree to which their effects may be considered non-conscious. Potential practical issues with applying such a framework are discussed, as are the implications for further research to inform the testing and development of interventions. A pragmatic means of conceptualising interventions targeted at non-conscious processes is a necessary prelude to testing the potency of such interventions. This can ultimately inform the development of interventions with the potential to shape healthier behaviours across populations.

The growing global burden of non-communicable disease (principally cancers, diabetes and cardiovascular disease) is largely determined by behaviours that are potentially modifiable, namely excessive consumption of food and alcohol, physical inactivity and smoking (WHO, 2014). Furthermore, these behaviours are socially patterned, being more common amongst those who are most socially deprived, thereby contributing to the increased morbidity and premature mortality observed in these groups (Stringhini et al., 2010). Yet such behaviours and their patterns have proven remarkably resistant to attempts to change them. Identifying interventions that are effective in changing health-related behaviours across populations and thereby reduce health inequalities arising from the social patterning of such behaviours is one of the foremost global public health challenges. It has been proposed that interventions that target non-conscious processes may prove effective in changing behaviour in populations, and potentially more so than interventions that principally engage conscious deliberative processes (Marteau, Hollands, & Fletcher, 2012). This important premise remains largely untested, however, hampered by the lack of a practicable conceptual framework necessary for characterising the processes by which interventions elicit behavioural responses. In this review, we outline a pragmatic approach to enable researchers to better describe the relative extent to which any given intervention to change health-related behaviours targets non-conscious processes, and thus begin to address these important issues.

Historically, the principal focus of non-regulatory approaches to changing health-related behaviour has been on information-based interventions (Marteau, Hollands, & Kelly, 2015). These interventions typically provide a persuasive message comprising verbal or numerical information to prompt individuals on the value or consequences of engaging in a given behaviour, leading to the formation of intentions to change that behaviour. They may also teach the skills necessary for change. Such approaches can provide the same generalised information to all members of a given population, for example, by using mass media to disseminate informative anti-smoking messages (Bala, Strzeszynski, Topor-Madry, & Cahill, 2013). Alternatively, they may use biological or genetic test information to provide personalised information to individuals about health risks attributable to dietary intake or physical inactivity. There is, however, an increasing recognition that many of these interventions are of limited effectiveness (Marteau et al., 2012). Whilst this observation is in accordance with evidence of limited intentional control of behaviour (Webb &Sheeran, 2006), it is important to clarify that this is not to dismiss the potential effectiveness of all interventions that purposefully engage conscious processes via, for example, providing persuasive information or facilitating problem solving or planning (including deliberative efforts to automate future responses to internal and external cues). Rather than being inherently ineffective, it may instead be that the content of such interventions is often inappropriately conceived or directed. Indeed, there is evidence for the effectiveness of a range of such interventions including smoking cessation programmes (West, May, West, Croghan, & McEwen, 2013), weight-loss programmes (Jebb et al., 2011) and implementation-intention interventions (Bélanger-Gravel, Godin, & Amireault, 2013) including when these are scaled up to population-level application (Neter, Stein, Barnett-Griness, Rennert, & Hagoel, 2014). However, the limited effectiveness that is often observed, particularly for predominantly information-based interventions, should prompt us to explore the potential of an additional approach, one that entails interventions that do not focus on engaging conscious deliberation via explicit communication, but instead target non-conscious processes occurring outside awareness.

Many processes determining our behaviour are non-conscious (Bargh & Morsella, 2008; Bargh, Schwader, Hailey, Dyer, & Boothby, 2012). It follows that interventions that target non-conscious processes may therefore prove effective in changing behaviour in populations (Marteau et al., 2012). Of particular interest are interventions that do not require individual delivery, targeting or instruction by those intervening, as these have the greatest potential to be readily scalable to the population level. Interventions meeting these criteria typically comprise changes to characteristics of the physical and social environments that surround us and shape our behaviour (Swinburn et al., 2011). Such interventions can be broadly classified as those that alter (i) the properties or (ii) the placement of external stimuli (see a recent typology of micro-environmental or choice architecture interventions (Hollands et al., 2013) and Supplemental material for further details and examples). These interventions do not require complex information to be understood in order for a behavioural response to be generated. Furthermore, as they are less dependent on levels of literacy, numeracy and self-regulatory capabilities, they may be particularly effective in those who are most socially deprived and who can be disadvantaged in such domains (Moffitt et al., 2011; Spears, 2010). Reflecting these considerations, there is growing interest across the fields of psychology, behavioural economics, neuroscience, public health and policy in the potency of external stimuli to change behaviour outside awareness (Cohen & Babey, 2012; Dolan, Hallsworth, Halpern, King, & Vlaev, 2010; Felsen, Castelo, & Reiner, 2013; Lisman & Sternberg, 2013; Marteau et al., 2012; Sheeran, Gollwitzer, & Bargh, 2013; Thaler & Sunstein, 2008). These ideas have also gained traction within government and policy circles worldwide (Nesterak, 2014).

Whilst prior literature has examined the processes by which health-related behaviour may be influenced outside of awareness (Cohen & Babey, 2012; Sheeran et al., 2013), despite high levels of interest and optimism, robust evidence of effectiveness of interventions that target non-conscious processes remains scarce. There are reports of interventions that impact on behaviour despite finding that participants are typically unaware of being exposed to the intervention (van Kleef, Otten, & van Trijp, 2012; Maas, de Ridder, de Vet, & de Wit, 2012; Papies & Hamstra, 2010). There are also a small number of systematic reviews and meta-analyses of interventions that could reasonably be assumed to operate at least partly outside of conscious awareness, such as altering food portion, package and tableware size (Hollands et al., 2015), that indicate important effects on behaviour. However, as was observed in Hollands et al.’s systematic review, participant awareness in intervention studies is often not assessed, or is minimally reported. Research efforts towards testing the important central premise – that interventions targeting non-conscious processes may have significant potential – and ultimately identifying and developing effective interventions, are hampered by the lack of a practicable conceptual framework necessary for coherent research characterising their effects. To examine whether interventions can reasonably be characterised as having potential to influence behaviour via targeting non-conscious processes, we first explore what is meant by conscious and non-conscious activation of behaviour by external cues or stimuli.

## Conceptualising conscious and non-conscious activation of behaviour

Determining whether any given behaviour can be described as conscious is a complex task. Each behaviour and its activation is a composite of many conscious and non-conscious processes and may arise from an array of internal and external cues and their interaction. This is further complicated by the fact that behaviours can be analysed and described at a number of levels and so the admixture of conscious and non-conscious processing may be different depending on the level at which the analysis is applied. For example, if I talk about a bicycle ride, my description may invoke the non-conscious processes that keep me balanced and moving, the conscious processes of deliberating where I am going and why, and a host of processes in between that may each in themselves be described at different levels. My route may be a well-known one that I may cycle automatically without deliberation, or it may be a novel one laboriously followed with a map. To take another example, of eating some potato chips, I may be aware of the endpoint of actually eating the chips, but not necessarily aware of why I am eating the chips. Rather than being a result of a premeditated decision, the behaviour may have been activated by an environmental cue such as a television advertisement, the influence of which I was unaware (Harris, Bargh, & Brownell, 2009). The key point here, and one to which we will return, is that, when we identify processes as conscious or non-conscious, we must be clear about the scale and level of analysis we are employing. We propose that by applying a consistent framework we can find an appropriate level to describe and analyse behaviours as a prelude to developing interventions aimed at changing them. The distinction between conscious and non-conscious behaviour may be informative in the context of developing ways to change population health behaviour because it reflects the degree of conscious deliberation that is required for a behaviour to be activated by a given intervention.

Conceptions of conscious and non-conscious behaviour can be framed in relation to dualisms that are present across the behavioural and brain sciences. Dual-process or dual-systems models of behaviour have received increasing attention in recent years within behavioural science and psychology (Hofmann, Friese, & Wiers, 2008; Sherman, Gawronski, & Trope, 2014; Strack & Deutsch, 2004). Such models propose two broad systems of human behaviour, one comprising actions towards identified goals resulting from reflective, reasoned processes, and the other comprising actions resulting from automatic associative processes cued by external stimuli. A number of terms has been applied to denote these two systems. We use the terms reflective and automatic to represent multiple, nuanced underlying components and conceptualisations. Although it is convenient to characterise reflective behaviour as conscious and automatic as non-conscious, Bargh (1994) and Moors and De Houwer (2006) deconstruct the meaning of automaticity, highlighting that this relates to multiple components or processes with varying degrees of overlap, each of which has been used to characterise automaticity. It is therefore more precise to consider these components individually. We focus here on the conscious–nonconscious dimension, which is widely invoked on its own terms and is also used to represent broader conceptions of automaticity in the behaviour change literature.

Within the behavioural neuroscience literature, a distinction is commonly made between habitual and goal-directed behaviour. Simply put, habitual responding entails an action prompted by the presence of a stimulus without the necessary representation of the goal of that action, the latter representation being a characteristic of goal-directed behaviour (see Gardner (2015) for a review of the habits literature as applied to health-related behaviour). This distinction provides a flexible, though simple, means of describing behaviours, but the question of how these phenomena relate to a distinction between conscious and non-conscious behaviour is complex. Lisman and Sternberg (2013) proposed that non-conscious behaviour equates to habitual behaviour, and conscious behaviour to non-habitual behaviour. Given that goal-directed behaviour is essentially characterised by the representation of a goal, this is a plausible idea. However, we suggest that, for practical purposes, it is more useful to consider both habitual and non-habitual behaviour as having the potential to be conscious or non-conscious depending upon the level of analysis. This is because, as already mentioned, any given behaviour comprises an admixture of conscious and non-conscious processes that may differ depending on the level at which analysis is applied. We provide here only a simplified overview of the literature on non-conscious processes and automaticity. More extensive literature reviews are available (Bargh et al., 2012; Moors & De Houwer, 2006).

## Key considerations in describing non-conscious activation of behaviour

**Figure 1.**
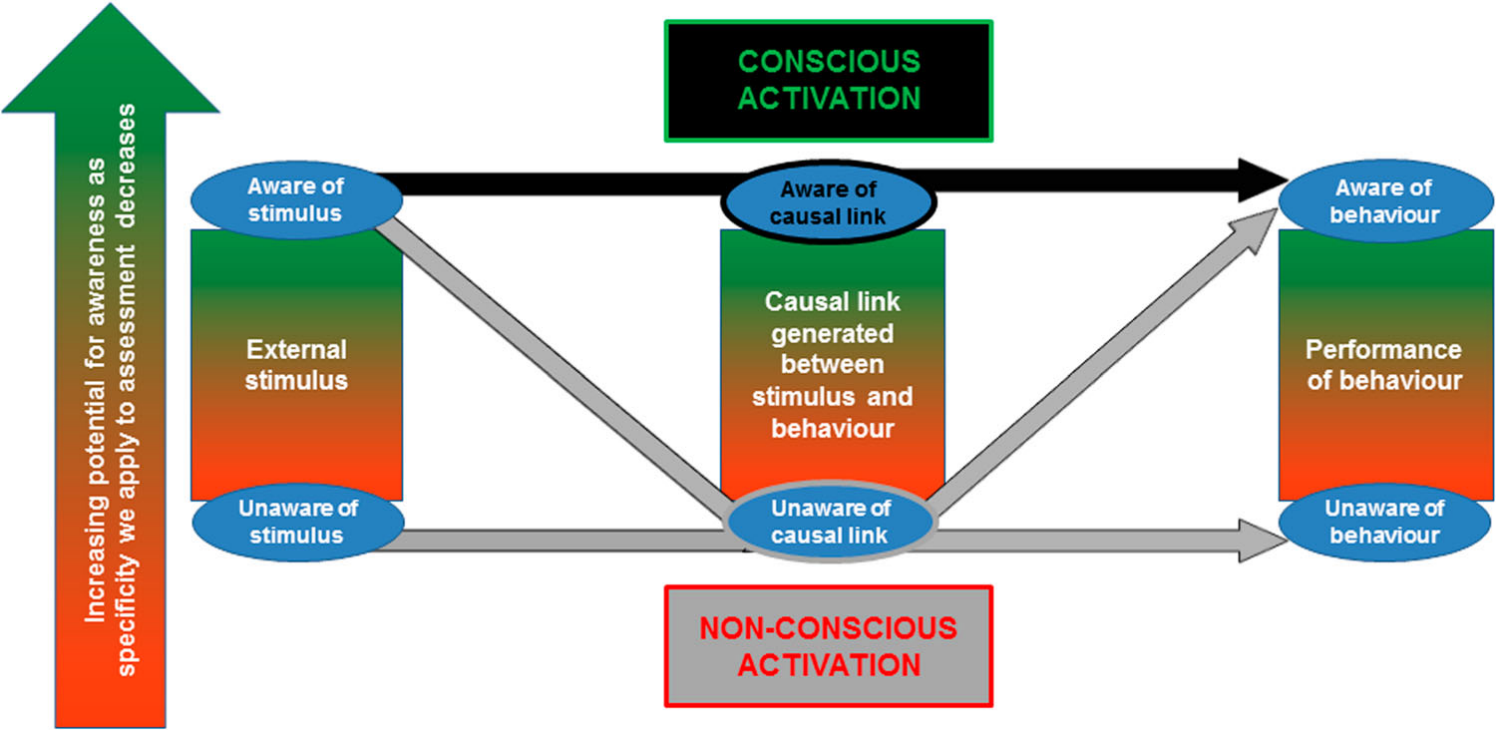
Conscious and non-conscious processes underlying behavioural activation cued by exposure to an external stimulus. The black path represents behavioural activation that would be regarded as conscious, whereby the actor is aware of a causal link between a stimulus and a behaviour. The grey paths represent a behaviour that would be regarded as non-conscious, whereby the actor is unaware of the causal link between a stimulus and a behaviour. The green–red shading represents the conscious–nonconscious spectrum, whereby awareness of each element is of a degree on a spectrum depending on the level at which awareness is analysed. Note: This figure is intended to provide a simple representation of behaviour activation and so does not include goal activation processes, but the principles apply equally to behaviours for which goals are represented and those for which they are not.

Figure 1 highlights two key premises that underpin our framework and merit further consideration: (i) A comprehensive analysis of a behaviour requires that we consider the enacted behaviour alongside the processes by which it is activated; and (ii) many levels of analysis are possible when describing a behaviour and awareness of the activation of behaviour.

### A comprehensive analysis of a behaviour requires that we consider the enacted behaviour alongside the processes by which it is activated

It has been suggested that if a person is able to report on an action then this constitutes conscious behaviour. Lisman and Sternberg (2013) apply the example of advertising, which, while it may trigger non-conscious processes, ultimately leads to conscious performance of a behaviour, such as buying a chocolate bar. They state that, ‘The fact that conscious decisions can be influenced by unconscious factors does not alter the fact that the decision itself is reportable and therefore conscious' (p. 278)*.* Whilst this criterion may appear initially reasonable and straightforward to apply, adopting it would nonetheless place considerable constraints on a comprehensive understanding of health-related behaviours. By focusing on the ultimate behavioural endpoint (such as buying a chocolate bar) and omitting consideration of the multiple processes that activated and shaped this behaviour, we sidestep important complexities and limit our ability to describe and explain it. We suggest that a more detailed examination of the processes by which a behaviour is activated can be helpful and so we should instead aim to consider the intermediary processes by which the cueing stimulus activates the response. Within this context, we propose, consistent with the position taken by other authors (Chartrand, 2005; Nisbett & Wilson, 1977), that conscious activation of behaviour comprises awareness of the causal link between the stimulus that activates the behavioural response and the performance of the behaviour. However, formation of such a causal inference that there was an effect of the stimulus on one's behaviour, logically requires awareness of both the external stimulus and of the ensuing behaviour. Therefore, the degree to which behaviour activated by external stimuli might be considered non-conscious is a function of the extent to which the actor is aware of the following elements:

- the external stimulus (i.e., the intervention);
- the ensuing behaviour; and
- the causal link between (a) the stimulus and (b) the behaviour

It is important to emphasise that our focus is on the activation of behaviour by external stimuli because our aim is to characterise differential awareness of the effects of external stimuli, in the form of interventions to change behaviour. For the sake of parsimony, we are also invoking a single external stimulus, but of course acknowledge that multiple external and also internal stimuli will often continue to be important determinants of behaviour irrespective of any intervention. Furthermore, people vary in terms of the stimuli to which they expose themselves and the effects thereof.

Whilst the theoretical position outlined here is not novel, our specific focus, upon translating these concepts for application to interventions to change health-related behaviour, is novel. We will now discuss each of the three elements above in turn. As illustration, throughout the following discussion we will draw on a set of exemplar intervention types (derived from Hollands et al., 2013) that have been implemented to shape healthier dietary behaviour: altering portion sizes of food (sizing) (Rolls, Roe, & Meengs, 2010); exposing shoppers to recipe posters to prime dieting goals (priming) (Papies & Hamstra, 2010); placing foods nearer or further away from people (proximity) (Maas et al., 2012); and altering the relative availability of healthy and unhealthy food options (availability) (van Kleef et al., 2012). These interventions are appropriate case studies as they have the potential to activate behaviour via non-conscious processes. They do not rely on conscious deliberation of explicit information and it is therefore less likely that the actor will be cognizant of, or reflect upon, their intended effects. This is demonstrated in the cited examples of each, in which participant awareness of the intervention to which they have been exposed has been assessed, revealing most to be unaware.

### Awareness of the external stimulus (i.e., the intervention)

Dehaene, Changeux, Naccache, Sackur, and Sergent (2006) discuss the characteristics of conscious processing with a focus on the perception of visual stimuli. But how does this translate to interventions that occur in real-world environments, where there may be a complex range of stimuli (not only visual) and differing across a range of properties or characteristics? One possibility is to define consciousness in relation to simple awareness of the presence versus absence of the external stimulus. In the case of portion sizing interventions, this would mean awareness of the presence of the food portion that was subject to manipulation. Because nearly all instances of naturally occurring stimuli are supraliminal (Bargh & Morsella, 2008), this places a relatively low threshold on the assessment of consciousness. As such, we would be highly likely to ascribe awareness of the stimulus if we used such a criterion. A more stringent test and a more appropriate threshold when thinking about changing behaviour is awareness of the specific properties or characteristics of the stimulus that comprise the intervention manipulation (the more detailed this is able to be, the more it is suggestive of increasing awareness). These levels of awareness can be determined on their own (absolute) terms, or in relative terms, as a difference relative to a prior exposure to the same or similar stimulus or environment. Which is more appropriate would depend on the nature of the implementation. In the example of portion size, this would mean awareness of the size or volume of the food portion (i.e., absolute), or awareness of a difference in size or volume of the food portion relative to a prior exposure in a comparable context (i.e., relative). For the previously cited example of priming, this may mean awareness of the poster or the recipe content therein; and for proximity and availability, respectively, the notable closeness or distance of the food or the notably large or small number of healthy or unhealthy food options that are available.

### Awareness of the ensuing behaviour

An actor's awareness of a given behaviour (e.g., eating food) can be characterised at a variety of levels. First, he or she may have no awareness that they have acted at all (e.g., not aware that they are eating or have eaten food subsequent to exposure to the stimulus). Second, he or she may be aware that they have acted in some way, but with limited awareness of the properties or characteristics of this behaviour. For example, they may be aware of eating or having eaten food but not aware of the amount of food consumed. Third, an actor may possess a higher level of awareness, which could be demonstrated either in absolute or relative terms. For example, they may be aware of eating a specified amount of food (absolute), or of eating a higher, similar or lower amount of food relative to typical or previous behaviour in a comparable context (relative).

A problem with trying to ascribe consciousness to a behaviour, even using a well-operationalised approach, is the complexity mentioned previously: any behaviour inevitably arises from a composite of a multitude of smaller, intermediary behaviours and processes, for which we could potentially assess awareness. For example, in the context of research on portion sizing interventions, likely outcomes of interest would be total consumption of, or total energy intake from, the food product which is subject to the intervention. However, if we wished, we could instead choose to focus on the speed of an individual's first approach to the food, or the amount they consumed in their first bite – and assess awareness correspondingly. In short, there comes a level of description where aspects of even the most reflective of behaviours fall below the level of awareness. A parallel can be drawn to the habits literature where it has been proposed that for complex behaviours it may be helpful to distinguish between the processes of (habitual) initiation of a behaviour and its performance (Gardner, 2015). However, what might seem a profound problem of characterising behaviour is of lesser concern in the context of a pragmatic approach to changing behaviour. Whilst, at a fine enough grain, any behaviour might be dismantled into non-conscious parts, the pertinent question is whether the level of analysis is relevant to the changes in behaviour we aim to elicit, before considering the question of awareness at that level. When working within the context of a behaviour change intervention we may often choose a higher-level behavioural endpoint, such as a selection or consumption behaviour, given the ultimate goal of demonstrating a change in behaviour that has some wider significance for health outcomes. There may also be contexts where a focus on a lower-level behaviour is equally or more relevant to the changes in behaviour that the intervention is intended to elicit, such as reducing bite size or speed of chewing (Shah et al., 2014).

### Awareness of the causal link between (a) the stimulus and (b) the behaviour

This criterion ultimately determines whether we can ascribe conscious activation of behaviour, but as illustrated in Figure 1, awareness of the causal link is logically predicated on an actor having some awareness of both the stimulus (the intervention) and of their resulting behaviour. If awareness of either of these components is absent then awareness of the causal link is inevitably also absent, and this would then be regarded as an example of non-conscious activation of behaviour. If awareness of both is present, but the actor remains unaware of a causal link, then again this would be regarded as representing non-conscious activation of the behaviour. As such, non-conscious behaviour in this context is characterised by a lack of awareness of the effect of external stimuli on one's behaviour (Bargh & Morsella, 2008). Conversely, if individuals are aware of all three elements then this would be regarded as an example of conscious activation of behaviour. Applying the example of portion sizing, an awareness of the causal link, and thus conscious activation of behaviour, would comprise a recognition that the characteristics of the behaviour (e.g., five sandwiches were eaten, or more sandwiches were eaten than usual) were influenced by the characteristics of the stimulus (e.g., the large number of sandwiches that was presented for consumption). Awareness is, however, a matter of degree and varies along a spectrum, meaning that individuals may recognise a causal link but misjudge its extent. As we now discuss, characterising the level of awareness also depends on the level of analysis that we apply.

### Many levels of analysis are possible when describing a behaviour and awareness of the activation of behaviour

Lisman and Sternberg (2013) propose that if a decision about a behaviour is reportable then it is conscious. This raises the question as to what precisely is meant by a ‘decision’ that is to be reported. Applying simple distinctions between awareness and a lack thereof may be relatively straightforward in a laboratory context, where awareness of a behaviour may refer to simply eating or not eating a food item presented to a participant where there are relatively few competing behavioural (or physical/spatial) possibilities. But if we take the example of buying a bottle of branded beer from a supermarket, how does Lisman and Sternberg's concept of conscious behaviour apply? Awareness of the behavioural decision is an ambiguous criterion because it could be applied at numerous levels of specificity or levels of explanation. It could mean awareness of several possible decisions, ranging from non-specific (such as the decision to take any amount of any alcoholic beverage from the shelf) to specific (such as the decision to take four bottles of a specific brand of bottled beer and none of any other brand). Alternatively, it could refer to different stages of activation of the behaviour, ranging from awareness of a prior realisation of wanting to purchase some beer at the supermarket, to awareness of placing bottles of beer in a shopping basket. Does awareness at any one of these points count as an equivalent demonstration of conscious activation of behaviour? We would argue that it does not and that it may be instructive to think in more nuanced terms about the nature of awareness of any given behaviour.

This example illustrates what we call the issue of specificity: awareness of behaviour and of its activation is a concept always understood relative to the level of specificity that is applied by the assessor. When we assess the degree of awareness of the activation of a behaviour, the more specific we are in our assessment of what constitutes awareness (i.e., the more detailed the knowledge we require from our interrogation), the less likely it is that the actor will be able to demonstrate awareness of the process, and thus the more likely we are to attribute any observed behaviour to a process of non-conscious activation. For example, if a person is presented with a large bowl of chocolate pudding, we might reasonably expect them to be aware whether they had consumed any of the pudding or not, but may not expect them to be aware of the precise absolute amount that they had consumed. Consequently, an inability to distinguish the amount being consumed may not represent a fundamental lack of awareness, but instead represent the difficulty of reporting such detailed objective information. We might, however, reasonably expect an adequate assessment of *relative* consumption, such as of having eating more or less pudding than usual.

In line with our prior comments concerning identifying an appropriate behavioural outcome, in assessing awareness of the activation of behaviour there is a need to apply a degree of specificity that is consistent with the aim of the specified intervention. Furthermore, if any assessment is to have the potential to aid in characterising behaviour by degree, it must possess the potential to detect differences in responses and so the threshold for ascribing awareness must not be set inappropriately low or high.

## Applying a framework of conscious and non-conscious activation of behaviour by an intervention

**Figure 2.**
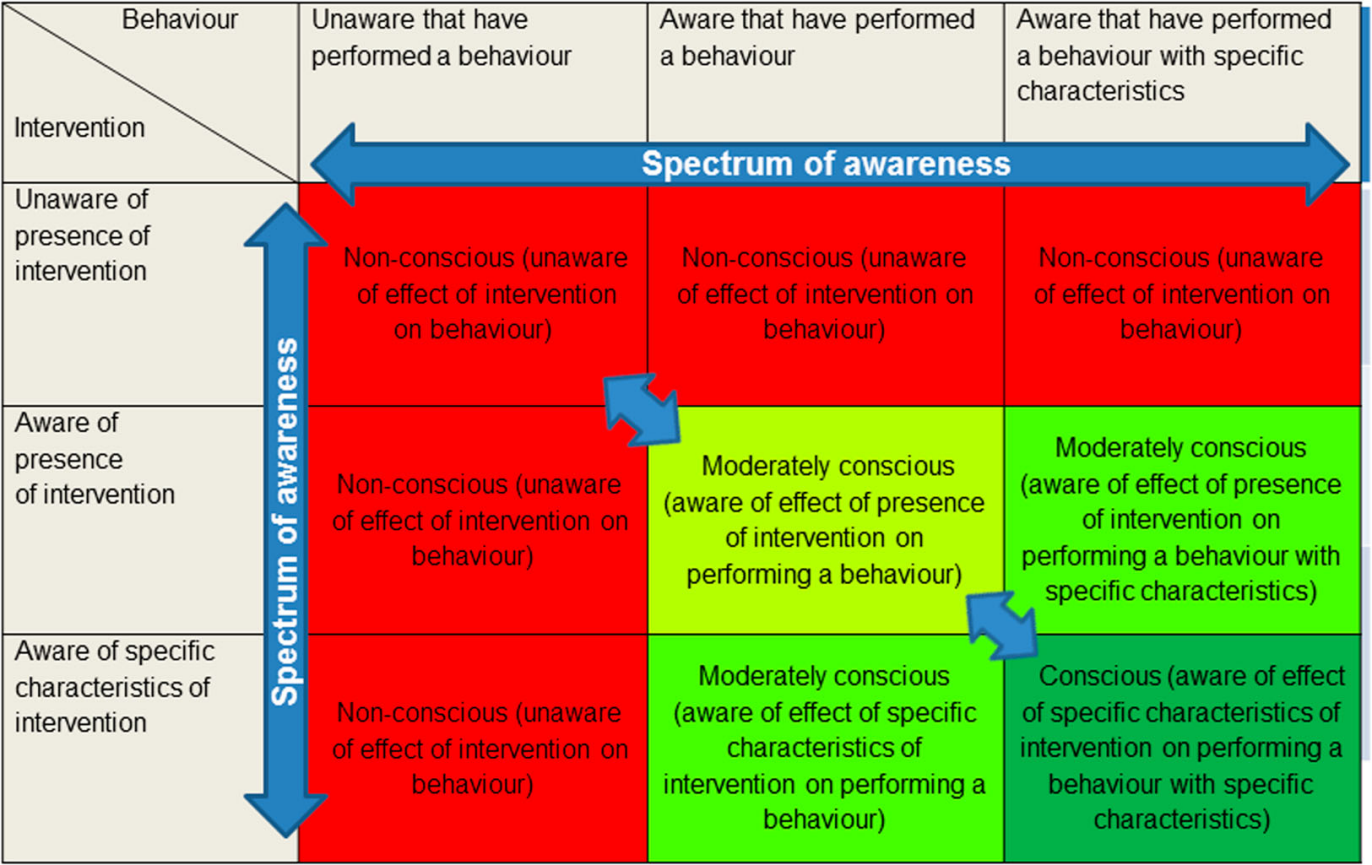
A framework of conscious and non-conscious activation of behaviour by an intervention. Conscious activation of behaviour (green) is characterised by awareness of both the intervention and the behavioural response. Non-conscious activation of behaviour (red) is characterised by a lack of awareness of the intervention and/or the behavioural response. Moderately conscious activation of behaviour (light green) indicates an assumed spectrum. For the purposes of this figure, we assume that, where there is some awareness of both intervention and behaviour components, an awareness of the causal link between these has also been generated to a varying degree (should this be absent, then the behaviour would inevitably be regarded as non-conscious).

In Figure 2, we present a framework applied to the context of interventions to change behaviour. This highlights that if the criteria we have outlined are consistently applied to such interventions, we can distinguish between relatively non-conscious and relatively conscious behaviour activation. It also indicates that these are broad and not discrete categories and awareness is most meaningfully viewed as occurring on a continuum.

Previously in this article, we assumed, for brevity and simplicity, an idealised situation in which we are able to assess the outlined criteria directly. Our concern, however, is with characterising interventions to change behaviour in real-world situations. We now consider how we may be able to characterise the extent to which any given intervention targets non-conscious processes, or in other words, is less reliant on engaging conscious deliberative processes. We can do this by applying the framework in Figure 2 to the effects of interventions. Awareness of the activation of behaviour can be assessed using various measurement approaches, although explicit measurement has been the predominant approach.

### Assessment of awareness of behaviour activation

Within the context of interventions or manipulations to change behaviour, the typical approach to operationalising the assessment of awareness of behaviour activation is to ask individuals directly. We regard self-report suggesting that an individual is unaware of the causal link between intervention stimuli and subsequent behaviour as a minimum necessary requirement for demonstrating that an intervention targets non-conscious processes. A funnel debriefing procedure may be employed by which individuals are asked increasingly specific questions about the nature of the intervention they have been exposed to and its potential for influencing their behaviour (Bargh & Chartrand, 2000). Participants may initially be asked if they are able to determine the broad purpose or hypotheses of the study, assuming that there is adequate blinding to this incorporated within the study design. Such general questions (e.g., ‘What do you think the study was about? What do you think the aim of the research is?’) are relevant in characterising the degree to which an intervention targets conscious engagement with its content. They allow assessment of whether participants recognise any potential link between the intervention and behaviour, for themselves or others, even if the intervention is ineffective. We may then move on to more specific questions to determine whether they noticed the characteristics of the stimuli that were presented, and whether they were aware of any link between these stimuli and their subsequent behaviour (e.g., ‘Did you notice anything special or notable about the (room/shop/restaurant/meal options/food you were given)?’; ‘Did anything (you were asked to do/you noticed in the room) affect your actions or how you were thinking?’) whilst being careful not to direct people towards specific responses. For these types of more specific questions, we would anticipate a lesser degree of awareness to be exhibited for a similarly effective intervention that targets non-conscious (versus conscious) processes.

If we again consider the example of portion sizing interventions, several researchers have assessed awareness in such a way. For example, Rolls et al. (2010) assigned participants to a series of differently sized portions of broccoli (as part of a meal) to see how this affected consumption. The researchers subsequently enquired about participants’ opinions of the purpose of the study but also whether they noticed any differences between the different experimental sessions. Despite this study employing a within-subjects design, making it more likely that the manipulated characteristics of the stimulus (i.e., the size of the portion) would be more salient, participants typically remained unaware of the purpose of the study and often of the experimental manipulation of portion size. In a priming intervention, Papies and Hamstra (2010) exposed participants to either a recipe poster displayed at a store entrance – an intervention designed to elicit dieting goals, or no poster – the control condition. When participants were asked whether they had noticed anything special about the store, fewer than 20% of participants in the intervention condition mentioned the recipe poster. This provides some support for characterising these interventions as having the potential to activate behaviour via non-conscious processes, as participants did not typically notice the experimental manipulations.

As highlighted in recent reviews and commentaries (Doyen, Klein, Simons, & Cleeremans, 2014; Hagger, Rebar, Mullan, Lipp, & Chatzisarantis, 2015; Labrecque & Wood, 2015; Newell & Shanks, 2014), assessing awareness of cognitive and behavioural processes via self-report has limitations. Doyen et al. (2014) identify a number of concerns, suggesting that verbal reports are inadequate for claiming processing without awareness. Our position on the use of such measures is as follows. First, we do not think that use of a self-report measure is invalidated because it fails to meet criteria proposed as necessary for documenting the complete absence of awareness. As these authors themselves assert, the need for documenting the complete absence of awareness depends on the claims that the researcher wishes to make. In research on behavioural interventions, we are ultimately interested in describing and developing more effective ways of changing behaviour. Understanding the underlying mechanisms is important in so far as it enables us to develop better interventions, but it is fundamentally a means to an end. We have no requirement to validate a theoretical position by definitively demonstrating the activation of non-conscious or conscious processes. Instead, by implementing the basic framework that we outline, we may be able to better characterise different types of interventions, by highlighting the variability in the degree to which they target non-conscious processes or to which they require conscious deliberation or engagement to change behaviour (at least as far as this is able to be assessed). This is the potential value that such an approach offers, even if this value largely remains to be demonstrated through the accretion of relevant data.

Second, we acknowledge that people are often unable (and at times unwilling) to report accurately on their own behaviour, and more so when a response is required after the fact (Nisbett & Wilson, 1977). Due to cognitive limitations and thus an inability to report relevant information, conscious report is imperfect and often rather limited in what it can capture. This means that we should not rely on detailed accounts of underlying mechanisms beyond the basic criteria we have outlined and, in determining and interpreting those criteria, apply a critical and cautious perspective. Even imperfect measures should, however, have the potential to fulfil the promise of the outlined approach, in at least enabling a broad mapping of relative levels of awareness between different interventions.

### Supplementary methods for assessing awareness

There may, however, be supplementary methods to self-report assessment that allow us to further corroborate or better characterise such an assessment. Bargh and Chartrand (2000) highlight the role of follow-up tests in corroborating the findings of funnel debriefing procedures, such as to determine actors’ ability to recognise and discriminate the stimuli they have been exposed to. It is not, however, immediately obvious to us how such approaches could be applied consistently to tests of interventions to change behaviour in real-world settings, where interventions may comprise a complex range of stimuli that differ across a range of properties. Alternative supplementary approaches may therefore be more adaptable.

First, we may employ implicit measures of cognition to assess the activation of cue-behaviour associative networks (Hagger et al., 2015). Instead of direct assessment via intentional report (e.g., questionnaire items), such measures assess processing via indirect assessment, such as response time tasks, that does not require awareness of the meaning of the response or the mental content that is being assessed (Nosek, Hawkins, & Frazier, 2011). If we observe that (a) such measures are affected by an intervention and this mediates the effect of the intervention on the behaviour; and (b) that measures that rely on direct reportability (such as questionnaire measures of reward value or behavioural intentions (Conroy, Hyde, Doerksen, & Ribeiro, 2010) are unaffected and do not mediate behavioural effects, then this provides additional detail for characterising the intervention as principally targeting non-conscious processes (though we cannot assume that the measures were comprehensive). This approach has been little applied to the study of interventions that are scalable to population-level, which is unsurprising given the practical challenges with gathering data on complex measures in a group intervention setting. It has, however, been used in controlled laboratory studies of behaviour change interventions (Hollands, Prestwich, & Marteau, 2011).

A second possible supplementary approach is to test whether, in the delivery of an intervention, adding explicit instructions to participants to encourage conscious deliberation on the mechanism of the intervention impacts on its effectiveness. For example, in a recent study (Cavanagh, Vartanian, Herman, & Polivy, 2014), participants were instructed regarding the way in which external influences, such as portion size, may affect food intake, including how to reduce such influences on their behaviour. This did not alter the effect of portion sizing on behaviour, that is, those given this instruction ate as much when provided with a larger portion as those not provided with this instruction. The fact that following the provision of such information, participants did not moderate the effect of the intervention is compatible with the effect of portion size on behaviour not being reliant on conscious engagement. This approach requires us to also apply the aforementioned explicit self-report approach; otherwise a possible interpretation is that the intervention is already working as a result of conscious engagement and so the additional explicit instructions will not affect this. A third, related approach is to test whether restricting one's ability to consciously engage with an intervention, for example, by simultaneously imposing a cognitive load, moderates the effect of an intervention. We would predict that if the effect of the intervention does not require conscious activation, then imposing a cognitive load would not significantly moderate it. However, we note that imposition of cognitive load may affect cognitive control of behaviour as well as conscious engagement. Further work is therefore needed to describe more precisely the cognitive resources needed for non-conscious behavioural control to ensure that these are not restricted in any attempt to restrict conscious behavioural control.

At present, it may be challenging to apply anything other than basic self-report measures of awareness to interventions implemented in the field, but we have aimed to highlight that there is value in applying pragmatic although imperfect methods. We do not, however, intend to deny the impetus to develop better approaches and more robust measures – challenges laid down should be regarded as a valuable catalyst towards increased precision and rigour in our methods, our reporting and in our theoretical interpretation. An important avenue of research will be the continued development of studies, observational and experimental, in more controlled environments that seek to examine, with temporal and spatial precision, the processes by which external cues are encountered and responded to. Such studies may valuably inform our understanding of both the mechanics (in terms of intervention and participant characteristics) of existing, conceptually similar interventions that are currently being applied in less controlled, real-world settings and also aid in the development of new interventions.

## Next steps

The key implication of the framework we have outlined is that those wanting to test and develop interventions that target non-conscious processes should consider attempting to characterise such processes by collecting primary data from intervention studies, alongside undertaking conceptual development work relating to methods of assessment and the mechanisms that they putatively represent. In this article, we propose what we regard to be a practicable starting point for this. We should also focus on improving our ability to characterise the nature and active components of interventions to change behaviour, in order to gain insight into why some interventions may elicit or require greater or lesser degrees of conscious engagement than others. As such, ongoing efforts to improve our understanding of the content, mechanisms and means of delivery of behaviour change interventions (Hollands et al., 2013; Michie et al., 2013) are complementary and should occur in parallel.

The central premise here is that interventions that rely less on conscious engagement and instead target non-conscious processes have significant potential for changing behaviour in populations. Increasing efforts to assess awareness would enable opportunities to begin testing this important premise which, if confirmed, would enhance the identification and development of effective interventions. First, at the aggregate, between-study level, it would enable the effectiveness of interventions in changing behaviour to be examined in relation to the degree to which they target non-conscious processes to change behaviour. Given sufficient primary data collection and accompanying conceptual developments, such an analysis could ultimately be possible within a systematic review framework. Second, at the more detailed within-study level, analysis could be conducted to examine the relationship between level of awareness of the intervention mechanism and its effects on behaviour. This would enable us to determine, for example, how the effect on behaviour differs between subgroups who are categorised as aware versus unaware (and whether the intervention remains effective when awareness is controlled for) and how closely subjective awareness of behaviour change maps on to actually observed changes in behaviour.

Finally, it would enable another key hypothesis (outlined in more detail elsewhere (Marteau et al., 2015)) to be examined, concerning the potential for behaviour change interventions to reduce health inequalities arising from the higher rates of unhealthier behaviours in more deprived groups. Because interventions that are less reliant on conscious, reflective engagement depend less on levels of literacy, numeracy and executive function, they may be particularly effective in those who are most socially and materially deprived and who can be disadvantaged in such domains. For example, there is evidence that person-centred behaviour change interventions that involve individual-level education and counselling may widen inequalities, whilst those that instead alter the environments that people are exposed to do not appear to do so (McGill et al., 2015). One could therefore examine whether interventions categorised as primarily targeting non-conscious processes are at least equally effective irrespective of how deprived the population they are applied to is, thus suggesting that their implementation would not further increase (and may even reduce) health inequalities.

Whilst our predominant focus in this article has been on interventions that do not target conscious deliberation and are instead more likely to activate behaviour outside awareness, our framework can also be applied to more reflective interventions, which we would expect to cluster on the opposing end of an assumed spectrum of awareness. Testing this assertion will also require the assessment of awareness in intervention contexts that we may currently assume work predominantly via conscious activation of behaviour. A final point is that, although we have framed this work primarily in relation to interventions to change health-related behaviour with the purpose of improving health, any developments in this area have the potential to inform other contexts in which the goal is to change behaviours across populations, such as pro-environmental behaviours to mitigate climate change (e.g., energy use and recycling), and consumer behaviours, where the purpose may be to change consumption in a way that may harm health.

## Conclusion

The framework we have outlined provides a basis for developing tests of our original premise, namely that interventions that target non-conscious processes and are less reliant on reflective, conscious engagement have significant potential for changing behaviour across populations. At this early stage of development, the potential value of the proposed framework rests principally in informing attempts to assess whether (a) awareness of the effects of interventions will indeed vary by degree, and that (b) this has implications for understanding and ultimately enhancing their effectiveness in changing behaviour. There are undoubtedly significant conceptual and practical challenges to be overcome, but we assert that these are outweighed by the potential benefits of such an approach. These include theoretical and methodological developments as well as, ultimately, interventions to change population behaviour that are both better understood and more effective.

## Supplementary Material

Supplemental_Material

## References

[CIT0001] Bala M. M., Strzeszynski L., Topor-Madry R., Cahill K. (2013). Mass media interventions for smoking cessation in adults. *Cochrane Database of Systematic Reviews*.

[CIT0002] Bargh J. A., Wyer R. S., Srull T. K. (1994). The four horsemen of automaticity: Awareness, intention, efficiency, and control in social cognition. *Handbook of Social Cognition*.

[CIT0003] Bargh J. A., Chartrand T. L., Reis H. T., Judd C. M. (2000). The mind in the middle: A practical guide to priming and automaticity research. *Handbook of research methods in social and personality psychology*.

[CIT0004] Bargh J. A., Morsella E. (2008). The unconscious mind. *Perspectives on Psychological Science*.

[CIT0005] Bargh J. A., Schwader K. L., Hailey S. E., Dyer R. L., Boothby E. J. (2012). Automaticity in social–cognitive processes. *Trends in Cognitive Sciences*.

[CIT0006] Bélanger-Gravel A., Godin G., Amireault S. (2013). A meta-analytic review of the effect of implementation intentions on physical activity. *Health Psychology Review*.

[CIT0007] Cavanagh K., Vartanian L. R., Herman C. P., Polivy J. (2014). The effect of portion size on food intake is robust to brief education and mindfulness exercises. *Journal of Health Psychology*.

[CIT0008] Chartrand T. L. (2005). The role of conscious awareness in consumer behavior. *Journal of Consumer Psychology*.

[CIT0009] Cohen D. A., Babey S. H. (2012). Contextual influences on eating behaviours: Heuristic processing and dietary choices. *Obesity Reviews*.

[CIT0010] Conroy D. E., Hyde A. L., Doerksen S. E., Ribeiro N. F. (2010). Implicit attitudes and explicit motivation prospectively predict physical activity. *Annals of Behavioral Medicine*.

[CIT0011] Dehaene S., Changeux J.-P., Naccache L., Sackur J., Sergent C. (2006). Conscious, preconscious, and subliminal processing: A testable taxonomy. *Trends in Cognitive Sciences*.

[CIT0012] Dolan P., Hallsworth M., Halpern D., King D., Vlaev I. (2010). *MINDSPACE: Influencing behaviour through public policy*.

[CIT0048] Doyen S., Klein O., Simons D. J., Cleeremans A. (2014). On the other side of the mirror: Priming in cognitive and social psychology. *Social Cognition*.

[CIT0013] Felsen G., Castelo N., Reiner P. B. (2013). Decisional enhancement and autonomy: Public attitudes towards overt and covert nudges. *Judgment and Decision Making*.

[CIT0014] Gardner B. (2015). A review and analysis of the use of ‘habit’ in understanding, predicting and influencing health-related behaviour. *Health Psychology Review*.

[CIT0015] Hagger M. S., Rebar A. L., Mullan B., Lipp O. V., Chatzisarantis N. L. D. (2015). The subjective experience of habit captured by self-report indexes may lead to inaccuracies in the measurement of habitual action. *Health Psychology Review*.

[CIT0016] Harris J. L., Bargh J. A., Brownell K. D. (2009). Priming effects of television food advertising on eating behavior. *Health Psychology*.

[CIT0017] Hofmann W., Friese M., Wiers R. W. (2008). Impulsive versus reflective influences on health behavior: A theoretical framework and empirical review. *Health Psychology Review*.

[CIT0018] Hollands G. J., Prestwich A., Marteau T. M. (2011). Using aversive images to enhance healthy food choices and implicit attitudes: An experimental test of evaluative conditioning. *Health Psychology*.

[CIT0019] Hollands G. J., Shemilt I., Marteau T. M., Jebb S. A., Kelly M. P., Nakamura R., Ogilvie D. (2013). Altering micro-environments to change population health behaviour: Towards an evidence base for choice architecture interventions. *BMC Public Health*.

[CIT0020] Hollands G. J., Shemilt I., Marteau T. M., Jebb S. A., Lewis H. B., Wei Y., Ogilvie D. (2015). Portion, package or tableware size for changing selection and consumption of food, alcohol and tobacco. *Cochrane Database of Systematic Reviews*.

[CIT0021] Jebb S. A., Ahern A. L., Olson A. D., Aston L. M., Holzapfel C., Stoll J., Caterson I. D. (2011). Primary care referral to a commercial provider for weight loss treatment versus standard care: A randomised controlled trial. *The Lancet*.

[CIT0022] van Kleef E., Otten K., van Trijp H. (2012). Healthy snacks at the checkout counter: A lab and field study on the impact of shelf arrangement and assortment structure on consumer choices. *BMC Public Health*.

[CIT0049] Labrecque J. S., Wood W. (2015). What measures of habit strength to use? Comment on Gardner (2015). *Health Psychology Review*.

[CIT0023] Lisman J., Sternberg E. J. (2013). Habit and nonhabit systems for unconscious and conscious behavior: Implications for multitasking. *Journal of Cognitive Neuroscience*.

[CIT0024] Maas J., de Ridder D. T. D., de Vet E., de Wit J. B. F. (2012). Do distant foods decrease intake? The effect of food accessibility on consumption. *Psychology & Health*.

[CIT0025] Marteau T. M., Hollands G. J., Fletcher P. C. (2012). Changing human behavior to prevent disease: The importance of targeting automatic processes. *Science*.

[CIT0026] Marteau T. M., Hollands G. J., Kelly M. P., Kaplan R. M., Spittel M., David D. H. (2015). Changing population behavior and reducing health disparities: Exploring the potential of “choice architecture” interventions. *Emerging Behavioral and Social Science Perspectives on Population Health*.

[CIT0027] McGill R., Anwar E., Orton L., Bromley H., Lloyd-Williams F., O'Flaherty M., Capewell S. (2015). Are interventions to promote healthy eating equally effective for all? Systematic review of socioeconomic inequalities in impact. *BMC Public Health*.

[CIT0028] Michie S., Richardson M., Johnston M., Abraham C., Francis J., Hardeman W., Wood C. E. (2013). The behavior change technique taxonomy (v1) of 93 hierarchically clustered techniques: Building an international consensus for the reporting of behavior change interventions. *Annals of Behavioral Medicine*.

[CIT0029] Moffitt T. E., Arseneault L., Belsky D., Dickson N., Hancox R. J., Harrington H., Caspi A. (2011). A gradient of childhood self-control predicts health, wealth, and public safety. *Proceedings of the National Academy of Sciences*.

[CIT0030] Moors A., De Houwer J. (2006). Automaticity: A theoretical and conceptual analysis. *Psychological Bulletin*.

[CIT0031] Nesterak E. (2014). Head of white house “nudge unit” Maya Shankar speaks about newly formed social and behavioral sciences team. *The Psych Report*.

[CIT0032] Neter E., Stein N., Barnett-Griness O., Rennert G., Hagoel L. (2014). From the bench to public health: Population-level implementation intentions in colorectal cancer screening. *American Journal of Preventive Medicine*.

[CIT0050] Newell B. R., Shanks D. R. (2014). Unconscious influences on decision making: A critical review. *Behavioral and Brain Sciences*.

[CIT0033] Nisbett R. E., Wilson T. D. (1977). Telling more than we can know: Verbal reports on mental processes. *Psychological Review*.

[CIT0034] Nosek B. A., Hawkins C. B., Frazier R. S. (2011). Implicit social cognition: From measures to mechanisms. *Trends in Cognitive Sciences*.

[CIT0035] Papies E. K., Hamstra P. (2010). Goal priming and eating behavior: Enhancing self-regulation by environmental cues. *Health Psychology*.

[CIT0036] Rolls B. J., Roe L. S., Meengs J. S. (2010). Portion size can be used strategically to increase vegetable consumption in adults. *The American Journal of Clinical Nutrition*.

[CIT0037] Shah M., Copeland J., Dart L., Adams-Huet B., James A., Rhea D. (2014). Slower eating speed lowers energy intake in normal-weight but not overweight/obese subjects. *Journal of the Academy of Nutrition and Dietetics*.

[CIT0038] Sheeran P., Gollwitzer P. M., Bargh J. A. (2013). Nonconscious processes and health. *Health Psychology*.

[CIT0039] Sherman J. W., Gawronski B., Trope Y. (2014). *Dual-process theories of the social mind*.

[CIT0040] Spears D. (2010). *Economic decision-making in poverty depletes behavioral control*.

[CIT0041] Strack F., Deutsch R. (2004). Reflective and impulsive determinants of social behavior. *Personality and Social Psychology Review*.

[CIT0042] Stringhini S., Sabia S., Shipley M., Brunner E., Nabi H., Kivimaki M., Singh-Manoux A. (2010). Association of socioeconomic position with health behaviors and mortality. *JAMA*.

[CIT0043] Swinburn B. A., Sacks G., Hall K. D., McPherson K., Finegood D. T., Moodie M. L., Gortmaker S. L. (2011). The global obesity pandemic: Shaped by global drivers and local environments. *Lancet*.

[CIT0044] Thaler R. H., Sunstein C. R. (2008). *Nudge: Improving decisions about health, wealth, and happiness*.

[CIT0045] Webb T. L., Sheeran P. (2006). Does changing behavioral intentions engender bahaviour change? A meta-analysis of the experimental evidence. *Psychological Bulletin*.

[CIT0046] West R., May S., West M., Croghan E., McEwen A. (2013). Performance of English stop smoking services in first 10 years: Analysis of service monitoring data. *BMJ*.

[CIT0047] WHO. (2014). *World Health Statistics 2014*.

