# Supplementary material for: Non-conscious processes in changing health-related behaviour: a conceptual analysis and framework
Source: Health Psychol Rev. 2016 Feb 16;10(4):381–94. doi: 10.1080/17437199.2015.1138093 (PMC5214381; doi:10.1080/17437199.2015.1138093)
Supplement: Supplemental_Material [file rhpr_a_1138093_sm7576.pdf]

APPENDIX 1

Examples of interventions that alter environmental stimuli to change health-related behaviours

We outline two broad types of intervention, in line with a classification described in a recent scoping review of relevant literature on the effect of micro-environmental or 'choice architecture' interventions on health-related behaviours (Hollands et al., 2013).

I. Interventions to alter the properties of external stimuli

Such interventions involve altering the design and sensory qualities of external stimuli (i.e. physical and social environments and the objects or stimuli contained within them) without explicitly communicating the intended message or information content. The non-conscious processes being actively targeted have been specified to varying degrees. Two sets of examples, which do not necessarily represent discrete mechanisms, are:

i) Interventions to activate or inhibit existing associations

The properties of external stimuli can be manipulated so that these environmental cues prompt behaviour through the activation or inhibition of existing associations or behavioural schema linked to the stimuli. Examples include: Exposing those entering shops to posters presenting food recipes to prime dieting goals outside of awareness, resulting in healthier purchasing and consumption (Papies & Hamstra, 2010; Papies, Potjes, Keesman, Schwinghammer, & van Koningsbruggen, 2014). Changes in table décor and seating arrangement have been used to influence individuals to consume amounts of food appropriate to either snack or meal occasions, an effect not mediated by their reported awareness of these characteristics (Shimizu, Payne, & Wansink, 2010). Reducing

exposure to alcohol cues in advertising decreases subsequent consumption of alcoholic drinks (Engels, Hermans, van Baaren, Hollenstein, & Bot, 2009). Finally, perceptual biases cause people to drink less from taller, narrower glasses than shorter, wider glasses (Wansink & Van Ittersum, 2003) and to consume alcohol at a slower rate from straight rather than curved glasses (Attwood, Scott-Samuel, Stothart, & Munafò, 2012).

ii) Interventions to alter existing associations or create new associations

External stimuli can be made more attractive so as to develop more positive associations. Examples include cosmetic improvements to stairwells to encourage stair use (Kerr, Yore, Ham, & Dietz, 2004) or enhancing the visual appeal of healthy foods by presenting them in an attractive and engaging way (Branen, Fletcher, & Hilbert, 2002). By contrast, stimuli can be made less attractive to develop negative associations. One example is altering the packaging of products to remove positive associations from branding elements, for example with plain packaging of cigarettes (Stead et al., 2013) or junk food (Robinson, Borzekowski, Matheson, & Kraemer, 2007). All these interventions can be conceptualized as increasing the reward value of healthier behaviour or decreasing the reward value of unhealthier behaviour.

II. Interventions to alter the placement of external stimuli

Such interventions involve altering the environment in such a way as to constrain or shape responses to make the least effortful course (physically and mentally) the most likely, leading to the preferred outcome of those intervening. The less effortful a behaviour is, the less likely it is to require conscious deliberation.

i) Interventions to alter proximity, accessibility, visibility or effort

Altering distances in environments to make it more and less effortful to engage in desired or unwanted behaviours makes the behaviours correspondingly less and more likely. For example,

moving areas dispensing energy-dense snack foods further away from, and those dispensing water or salad closer to patrons in cafeterias, can increase the healthiness of selected foods and beverages (Just & Wansink, 2009; Rozin et al., 2011)). Similarly, foods placed nearer are more likely to be eaten, with increased intake when placed 20cm away as opposed to 70cm or 140cm (Maas, de Ridder, de Vet, & de Wit, 2012). Changing the positions of items on menus increases the selection of those most immediately visible (Wisdom, Downs, & Loewenstein, 2010). Products are less likely to be selected when made less visible by placing them out of immediate sight, such as placement of cigarettes behind a cover or under the counter (Scheffels & Lavik, 2013), or behind transparent or opaque materials (Wansink, Painter, & Lee, 2006). Finally, stair use is encouraged by making the elevator a more effortful option by slowing down the speed at which it operates (VanHouten, Nau, & Merrigan, 1981).

ii) Interventions to alter availability

Increasing or decreasing the availability of a product or option for behaviour within an environment increases or decreases the effort required to engage with it. For example, increasing the number of available healthier food options in a restaurant or within a vending machine increases the likelihood that healthier foods are selected (van Kleef, Otten, & van Trijp, 2012). Reducing the number of available escalators increases the likelihood that people use the alternative of the stairs (Faskunger, Poortvliet, Nylund, & Rossen, 2003).

APPENDIX REFERENCES

Attwood, A. S., Scott-Samuel, N. E., Stothart, G., & Munafò, M. R. (2012). Glass Shape Influences Consumption Rate for Alcoholic Beverages. *PLoS ONE, 7*(8), e43007.

Branen, L., Fletcher, J., & Hilbert, L. (2002). Snack consumption and waste by preschool children served "cute" versus regular snacks. *Journal of Nutrition Education and Behavior, 34*(5), 279-282.

Engels, R. C. M. E., Hermans, R., van Baaren, R. B., Hollenstein, T., & Bot, S. M. (2009). Alcohol Portrayal on Television Affects Actual Drinking Behaviour. *Alcohol and Alcoholism, 44*(3), 244-249.

Faskunger, J., Poortvliet, E., Nylund, K., & Rossen, J. (2003). Effect of an environmental barrier to physical activity on commuter stair use. *Scandinavian Journal of Nutrition, 47*(1), 26-28.

Hollands, G. J., Shemilt, I., Marteau, T. M., Jebb, S. A., Kelly, M. P., Nakamura, R., . . . Ogilvie, D. (2013). Altering micro-environments to change population health behaviour: towards an evidence base for choice architecture interventions. *BMC Public Health, 13*(1), 1218.

Just, D., & Wansink, B. (2009). Smarter lunchrooms: Using behavioral economics to improve meal selection. *Choices, 24*(3), 1-7.

Kerr, N. A., Yore, M. M., Ham, S. A., & Dietz, W. H. (2004). Increasing stair use in a worksite through environmental changes. *American Journal of Health Promotion, 18*(4), 312-315.

Maas, J., de Ridder, D. T. D., de Vet, E., & de Wit, J. B. F. (2012). Do distant foods decrease intake? The effect of food accessibility on consumption. *Psychology & Health, 27,* 59-73.

Papies, E. K., & Hamstra, P. (2010). Goal priming and eating behavior: Enhancing self-regulation by environmental cues. *Health Psychology, 29*(4), 384-388.

Papies, E. K., Potjes, I., Keesman, M., Schwinghammer, S., & van Koningsbruggen, G. M. (2014). Using health primes to reduce unhealthy snack purchases among overweight consumers in a grocery store. *International Journal of Obesity, 38*(4), 597-560.

Robinson, T. N., Borzekowski, D. L. G., Matheson, D. M., & Kraemer, H. C. (2007). Effects of Fast Food Branding on Young Children's Taste Preferences. *Archives of Pediatrics & Adolescent Medicine, 161*(8), 792-797.

Rozin, P., Scott, S., Dingley, M., Urbanek, J. K., Jiang, H., & Kaltenbach, M. (2011). Nudge to nobesity I: Minor changes in accessibility decrease food intake. *Judgment and Decision Making, 6*(4).

Scheffels, J., & Lavik, R. (2013). Out of sight, out of mind? Removal of point-of-sale tobacco displays in Norway. *Tobacco Control, 22*, e37-e42

Shimizu, M., Payne, C. R., & Wansink, B. (2010). When snacks become meals: How hunger and environmental cues bias food intake. *International Journal of Behavioral Nutrition and Physical Activity, 7*(1), 1-6.

Stead, M., Moodie, C., Angus, K., Bauld, L., McNeill, A., Thomas, J., . . . Bryce, S. L. (2013). Is Consumer Response to Plain/Standardised Tobacco Packaging Consistent with Framework Convention on Tobacco Control Guidelines? A Systematic Review of Quantitative Studies. *PLoS ONE, 8*(10), e75919.

van Kleef, E., Otten, K., & van Trijp, H. (2012). Healthy snacks at the checkout counter: A lab and field study on the impact of shelf arrangement and assortment structure on consumer choices. *BMC Public Health, 12*(1), 1072.

VanHouten, R. V., Nau, P. A., & Merrigan, M. (1981). Reducing elevator energy use: A comparison of posted feedback and reduced elevator convenience. *Journal of Applied Behavior Analysis, 14*(4), 377-387.

Wansink, B., Painter, J. E., & Lee, Y.-K. (2006). The office candy dish: proximity's influence on estimated and actual consumption. *International Journal of Obesity, 30*(5), 871-875.

Wansink, B., & Van Ittersum, K. (2003). Bottoms up! The influence of elongation on pouring and consumption volume. *Journal of Consumer Research, 30*(3), 455-463.

Wisdom, J., Downs, J. S., & Loewenstein, G. (2010). Promoting healthy choices: Information versus convenience. *American Economic Journal: Applied Economics*, 164-178.
